# Dynamics of directional tuning and reference frames in humans: A high-density EEG study

**DOI:** 10.1038/s41598-018-26609-9

**Published:** 2018-05-29

**Authors:** Hirokazu Tanaka, Makoto Miyakoshi, Scott Makeig

**Affiliations:** 1 School of Information Science Japan, Advanced Institute of Science and Technology, 1-1 Asahidai, Nomi, Ishikawa, 923-1292 Japan; 2 Swartz Center for Computational Neuroscience, Institute of Neural Computation University of California San Diego, 9500 Gilman Drive # 0559, La Jolla, CA 92093-0559 USA

## Abstract

Recent developments in EEG recording and signal processing have made it possible to record in an unconstrained, natural movement task, therefore EEG provides a promising approach to understanding the neural mechanisms of upper-limb reaching control. This study specifically addressed how EEG dynamics in the time domain encoded finger movement directions (directional tuning) and posture dependence (movement reference frames) by applying representational similarity analysis. High-density EEG covering the entire scalp was recorded while participants performed eight-directional, center-out reaching movements, thereby allowing us to explore directional selectivity of EEG sources over the brain beyond somatosensory areas. A majority of the source processes exhibited statistically significant directional tuning during peri-movement periods. In addition, directional tuning curves shifted systematically when the shoulder angle was rotated to perform the task within a more laterally positioned workspace, the degree of tuning curve rotation falling between that predicted by models assuming extrinsic and shoulder-based reference frames. We conclude that temporal dynamics of neural mechanisms for motor control can be studied noninvasively in humans using high-density EEG and that directional sensitivity of motor and non-motor processing is not limited within the sensorimotor areas but extends to the whole brain areas.

## Introduction

Control of voluntary movements involves a series of computations including coordinate transformation and motor planning, execution, and evaluation. Neuron-scale brain mechanisms underlying such computations have been investigated in electrophysiological experiments with behaving monkeys. Neural activities during reaching movements have been recorded from both cortical and subcortical motor areas. A notable finding is that neural spike rates in motor-related areas exhibit broad, cosine-like extrinsic tuning with respect to movement direction, a property known as directional tuning. Directional tuning was first reported in primary motor cortex^1^ and subsequently in premotor cortex^2^, supplementary motor cortex^3^, pre-parietal Brodmann Area 5^2^, parietal reach region^4^, and in cerebellum^5^. The fact that directional tuning is not limited within the primary motor cortex suggests that directional tuning is related to not only pure motor actions but also motor preparation, visuomotor transformation or sensorimotor integration. In this study we use directional tuning in this broad sense. A preferred direction of a neuron (the movement direction at which the neuron spikes most rapidly) may or may not change with changes in posture or workspace geometry^6,7^, indicating either intrinsic or extrinsic reference frames. For wrist movements performed using pronated, midway, or supinated postures, for example, neural spike profiles consistent with extrinsic-like and muscle-like reference frames have both been found in primary motor cortex^8^ whereas only an extrinsic-like reference frame was consistent with spike tuning observed in ventral premotor cortex^9^.

Little is known about single-neuron mechanisms of voluntary movements in humans because invasive, single-unit recording from human subjects is restricted to activity recorded for clinical diagnostic or surgery planning purposes. Therefore, most studies on humans have employed non-invasive neuroimaging methods such as using fMRI to measure BOLD signal effects^10–13^. However, these fMRI studies have two limitations. First, only small movements have been examined due to limited space inside an MRI scanner, directional tuning changes associated with changes in movement posture have not yet been studied. Second, because of the inherently low pass and delayed nature of BOLD, rapid, sub-second dynamics of neural activities occurring at the speed of motor acts themselves cannot be resolved. Due to these limitations, dynamics and posture dependence of directional tuning have not been studied in the previous fMRI studies.

EEG is an imaging modality with temporal resolution of milliseconds and imposes little movement constraints, so it has been increasingly applied to human voluntary movements and brain-computer interfaces. EEG and ECoG recordings have been employed to investigate movement-related parameters from signals in the spectro-temporal domain in the sensorimotor areas. Most of previous approaches have tackled the problem of neural encoding of movement parameters by decoding various parameters of interest from EEG signals or spectro-temporal components in the sensorimotor areas, including movement directions^14–16^, movement trajectories^17^, movement types^18^, movement onsets^19^, and EMG^20,21^. These previous studies, however, focused mostly on decoding from the sensorimotor areas, and how movements are encoded in other areas of the brain is still largely unexplored. More importantly, experimental designs in these studies were not directly comparable to those used in the monkey electrophysiology studies.

It is thus desirable to employ larger-scale brain recording methods to build a holistic understanding of how movements are represented over the whole brain. Recent breakthroughs in physiological measurement hardware, signal processing, and computational modeling and statistics have enabled robust non-invasive EEG recordings with no or little constraint on body movements, an imaging advance known collectively as Mobile Brain/Body Imaging or MoBI^22,23^. This study exploited the MoBI techniques to investigate how movement parameters are encoded over the whole brain in an exploratory way. We specifically addressed directional tuning and reference frames during upper-limb movements because these are the properties that have been extensively studied in primate electrophysiology as reviewed above. The advantage of EEG recording over whole scalp is to make it possible to examine movement directions and reference frames over the brain without restricting to the somatosensory areas. We therefore took an exploratory approach to resolve EEG sources by independent component analysis and to assess directional tuning and reference frames by representational similarity analysis.

## Results

### Movement kinematics

**Figure 1:**
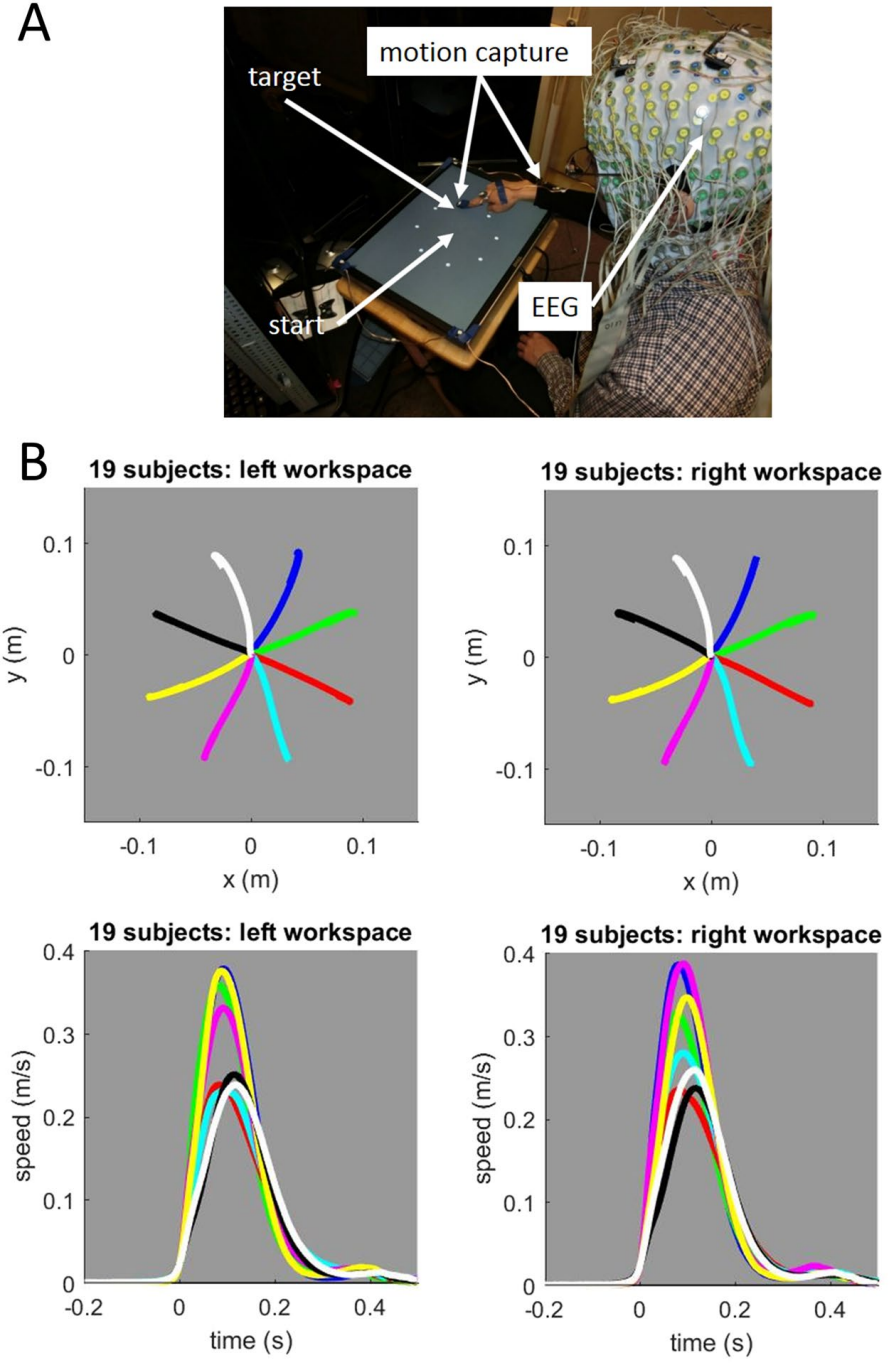
(**A**) Experimental setup. (**B**) Kinematics of finger movements (paths and velocity profiles) in two workspaces. Kinematics was averaged over all trials except “too early” or “too late” trials in which movement onset was more than 300 ms before or the Go event.

19 Subjects performed center-out reaching movements to one of eight targets either in the left or right workspace (see *Pointing task* in *Methods*) while their EEG and finger movements were recorded (*Simultaneous recording of high-density EEG and body motion* in *Methods*) (Fig. 1A). On average 1348 (SD 266) trials per subject had motion onsets within 300 ms of the Go events and were retained for the further analysis (see *Motion data analysis* in *Methods*). Recorded finger movement trajectories were mostly straight. Velocity profiles were single-peaked (Fig. 1B), as reported for both monkeys and humans. Movements toward targets near 45° and 225° were faster, and movements toward targets near 135° and 315° slower, reflecting upper limb biomechanical differences. Average movement duration was 253 ± 56 SD ms and 248 ± 53 SD ms in the left and right workspaces, respectively.

### EEG

We have applied independent component analysis to resolve EEG sources (*Independent component analysis* in *Methods*). Data cleaning during preprocessing rejected 11.2 ± 7.4 SD channels and 24 ± 17 SD % of trials; after channel and trial rejection, 367 ICs from the 19 subjects remained for further selection. Those components accounting for artifact from eye movements, blinks, and muscle activity were visually identified according to their characteristic spectra and component maps and excluded. In this manner, we obtained 267 ICs for the final analyses. Equivalent dipole locations for these brain-based ICs were distributed relatively uniformly over the cortical surface, excepting in the left frontal area (*Estimating equivalent current dipoles for EEG sources* in *Methods*).

### Clusters of independent dipolar sources in group data

**Figure 2:**
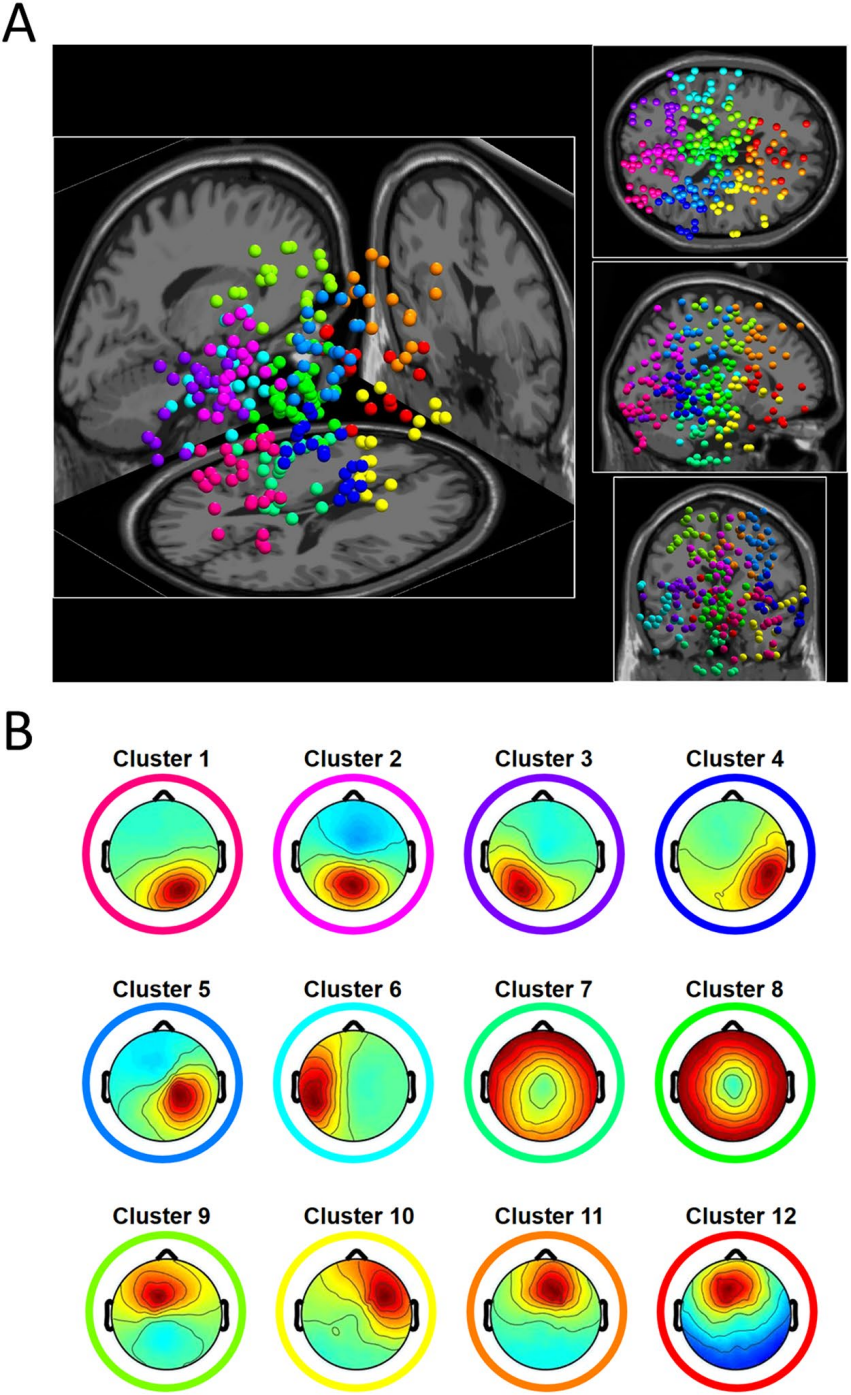
(**A**) 259 independent EEG sources classified into twelve clusters. EEG sources with the same color belong to the same cluster. (**B**) Average component projection maps for the 12 clusters, encircled with the same colors used in Panel A.

The equivalent dipolar sources from all subjects were grouped together and classified into twelve clusters based on their dipole locations and spectra (*Group analysis* in *Methods*). Eight of the 267 sources entered into the clustering were identified as outliers and not included in further analyses. The 12 component clusters were numbered according to the positions of their centroids along the anterior-posterior axis (Fig. 2A), and a mean component map for each cluster was obtained by averaging individual maps of components in each cluster (Fig. 2B). Each cluster contained on average 21.6 dipolar sources from 13.7 subjects on average. The centroids and the numbers of dipoles of these clusters are summarized in Table 1, and the centroid locations are summarized in Supplementary Figure 7.

**Table 1:** Details of twelve clusters.

Cluster number	#Subjects	#Dipolar sources	#Dipolar sources with statistical significant directional tuning	C.O.M. position (MNI)
1	16	26	18	[24, −82, −19]
2	15	19	14	[−1, −64, 30]
3	15	21	10	[−33, −64, 0]
4	13	20	4	[47, −46, −6]
5	14	21	8	[31, −33 44]
6	16	20	5	[−52, −30, −14]
7	12	21	10	[−4, −29, −45]
8	12	35	10	[−2, −23, −2]
9	13	23	12	[−21, −15, 50]
10	10	18	5	[41, −1, −21]
11	13	18	4	[15, 20, 42]
12	15	17	8	[−6, 25, −9]

### Within-workspace direction tuning widths

**Figure 3:**
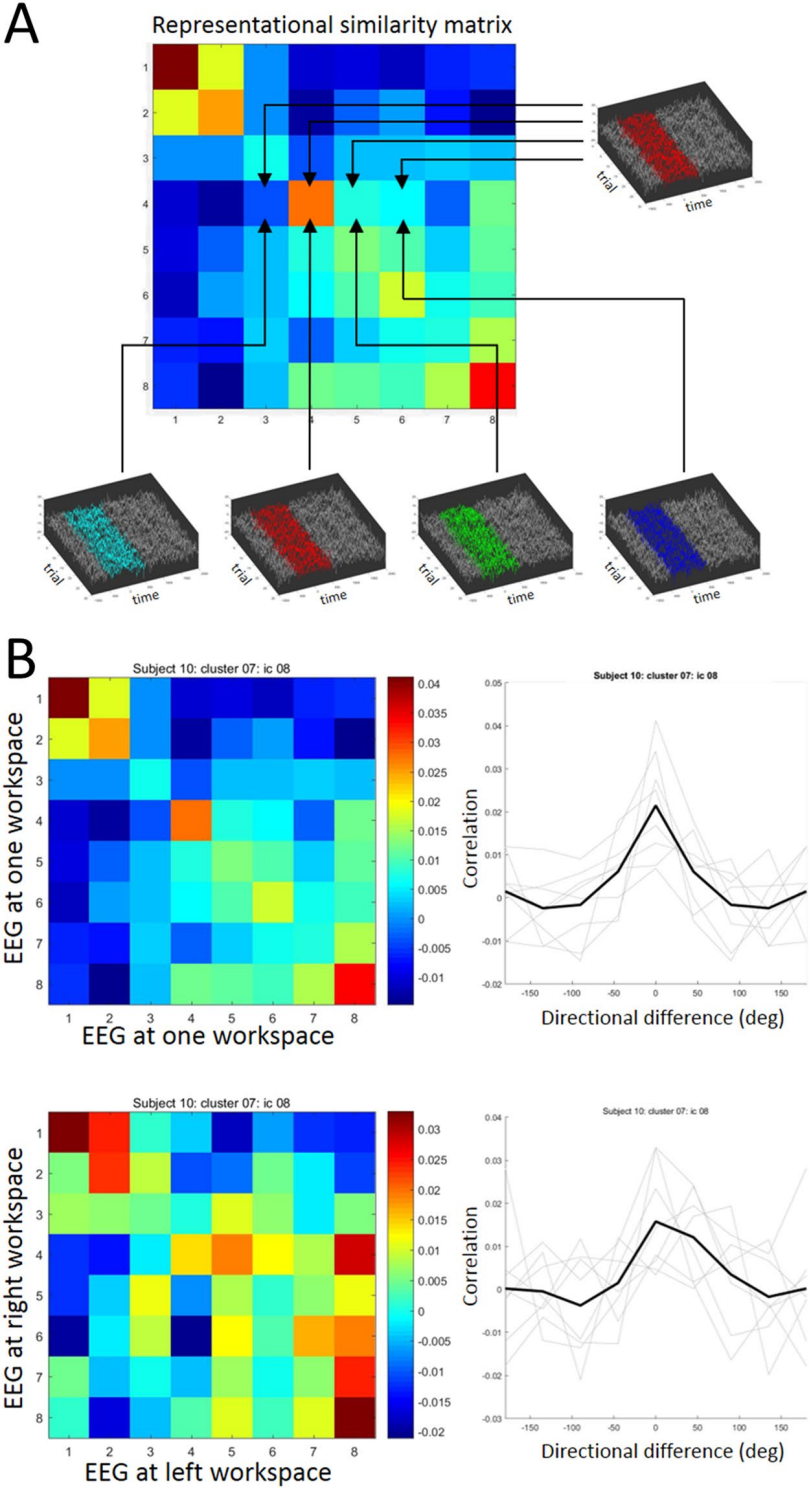
Computation of directional tuning and tuning shifts between workspaces. (**A**) Computation of a representation similarity matrix whose (*i*, *j*) components are computed as the Pearson correlation coefficient between all pairs of epochs time locked to movements in directions *i* and *j*. Tuning curves from the matrices computed (**B**) within one workspace and (**C**) across two workspaces.

**Figure 4:**
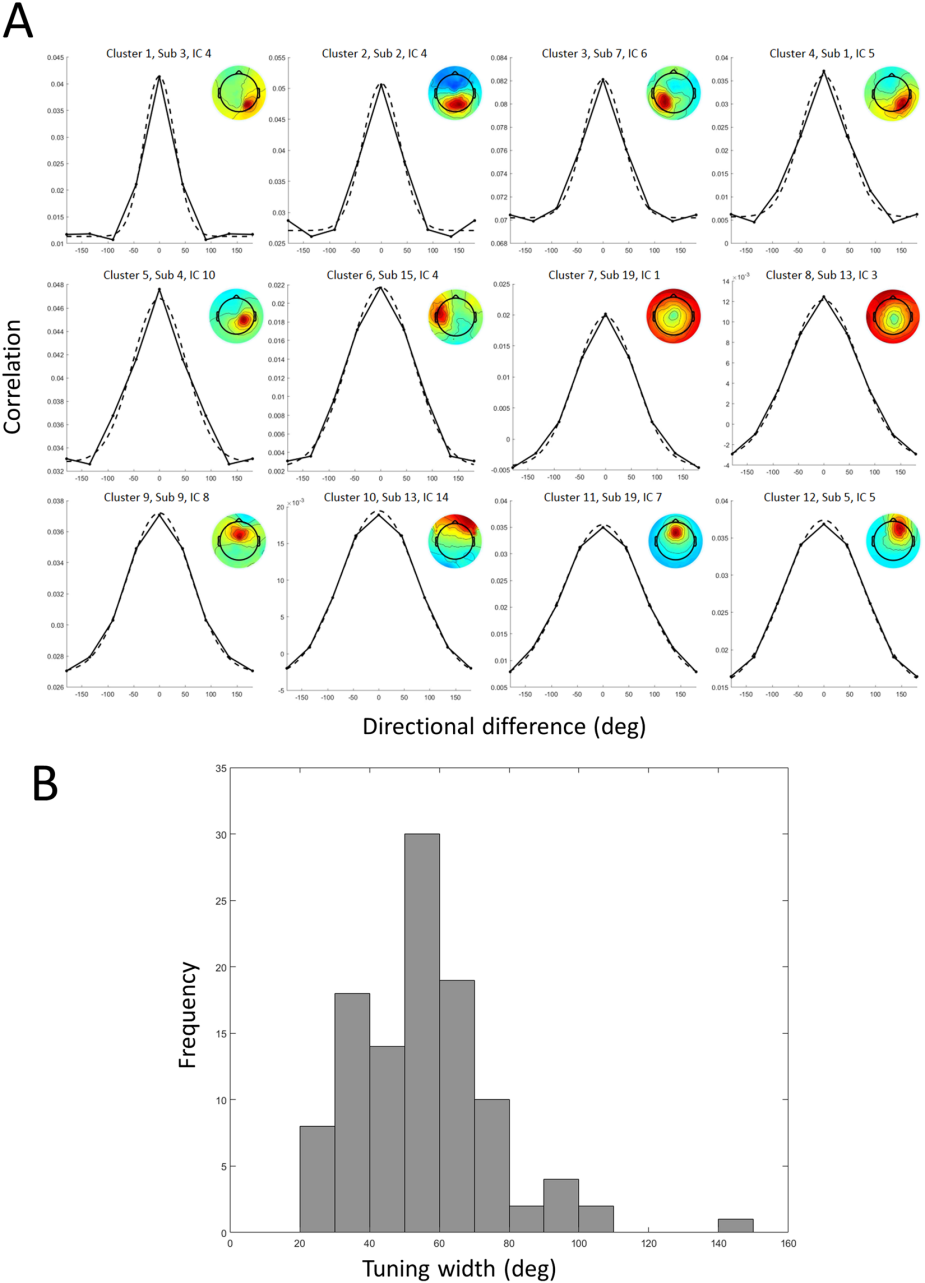
(**A**) Directional tuning of representative dipoles from twelve clusters. Each tuning curve (solid) is fitted with a Gaussian function (dashed), with a corresponding component map. (**B**) Histogram of directional tuning widths from all components that exhibited statistically significant directional tuning.

**Figure 5:**
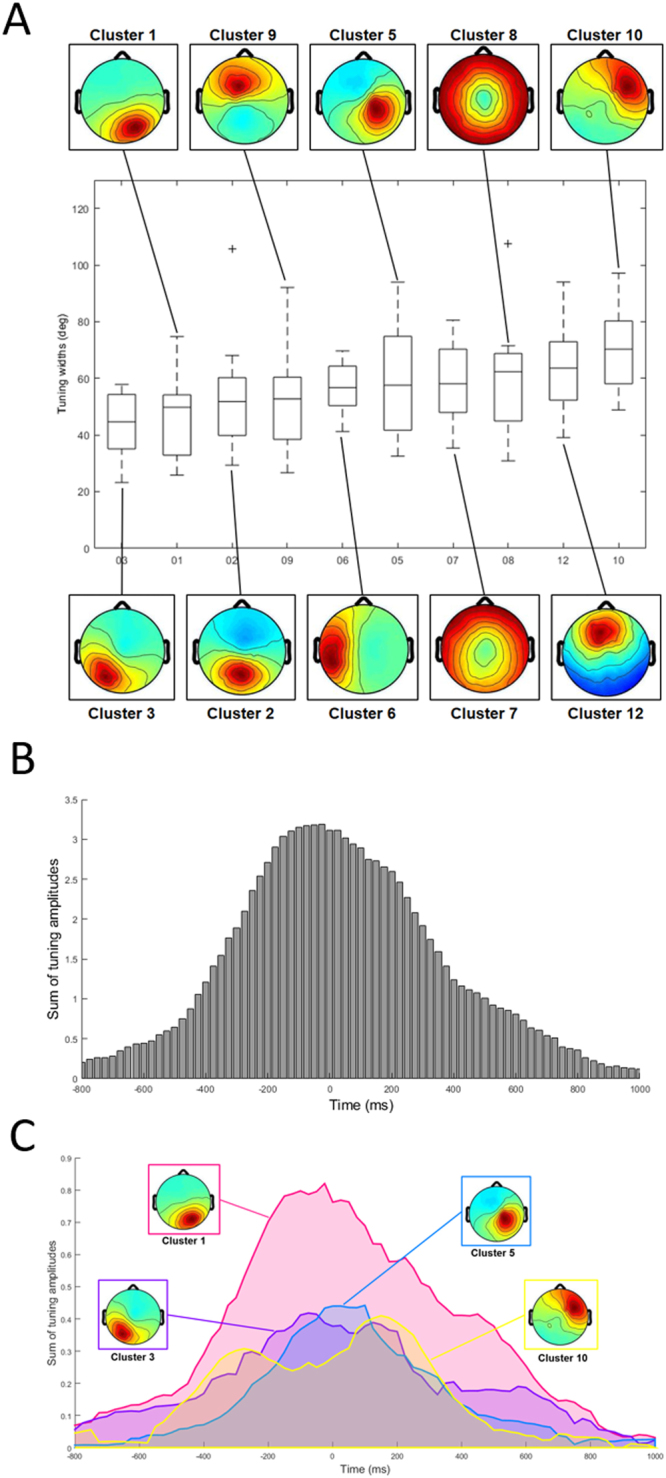
(**A**) Widths of directional tuning found in twelve clusters, sorted in an ascending order of the median values. For visualization, clusters that contained five or more dipole sources are included. (**B**) Temporal changes of directional tuning from −800 ms to 1000 ms. Each bin represents a sum of correlation amplitudes of dipoles in each time window. (**C**) Temporal changes of directional tuning of four clusters (1, 3, 5 and 10) which exhibited maximal peak values.

Directional tuning was computed for all dipolar sources by representational similarity analysis in the latency window from −250 ms to +250 ms surrounding movement onsets in one workspace (right or left) (*Computation of directional tuning within a single workspace* in *Methods*, see also Fig. 3). For further analyses, we identified 161 ICs whose directional tuning fit a Gaussian with *R*^2^ > = 0.9; among the 161 ICs, 108 IC exhibited statistically significant height above the 95% threshold (*Statistical testing* in *Methods*). The numbers of ICs with statistical directional tuning in each cluster were detailed in Table 1. Examples of tuning curves obtained from representative dipolar sources of twelve clusters are presented in Fig. 4A. Clearly, most dipolar sources exhibited single-peaked, unimodal directional tuning curves that could be well fit by a Gaussian function, indicating that our method of computing directional tuning could capture direction-by-direction similarity of IC time courses. Their tuning widths range from narrow to broad (most between 30° and 100°) (Fig. 4B). Statistically significant directional tuning was not limited to motor-related cortical areas but was quite broadly distributed over the brain. To investigate whether tuning width varied systematically between clusters, mean direction tuning width for each cluster was computed and these were compared across clusters (Fig. 5A). This cluster-by-cluster analysis showed that direction tuning was narrower in posterior clusters and broader in anterior clusters, although variation in tuning width within clusters was relatively large.

Next, to examine the temporal dynamics of tuning, direction tuning curves were computed for 500-msec sliding latency windows (−1000 to + 1000 ms relative to movement onsets), advanced in 25-ms steps. We characterized the strength of directional tuning by computing a sum of tuning amplitudes in each time window (Fig. 5B). Clearly, directional tuning exhibited a peak around the movement onset. To investigate which clusters contributed to directional tuning, the temporal change and peaks of tuning amplitudes were computed cluster by cluster. Among the twelve clusters, four clusters (#1, #3, #5 and #10) were identified to have maximal peak values of directional tuning amplitudes (Fig. 5C). The occipital clusters (1 and 3) had earlier peaks at about 100 ms before the movement onset, followed by the parietal cluster (#5) peaked around the movement onset, indicating that certain information of movement direction might have transferred from the occipital to the parietal areas. Interestingly, the frontal cluster (#10) had bimodal peaks of direction tuning amplitudes at about 275 ms before and 150 ms after the movement onset, suggesting that this cluster might have been involved in both movement preparation and execution.

**Figure 6:**
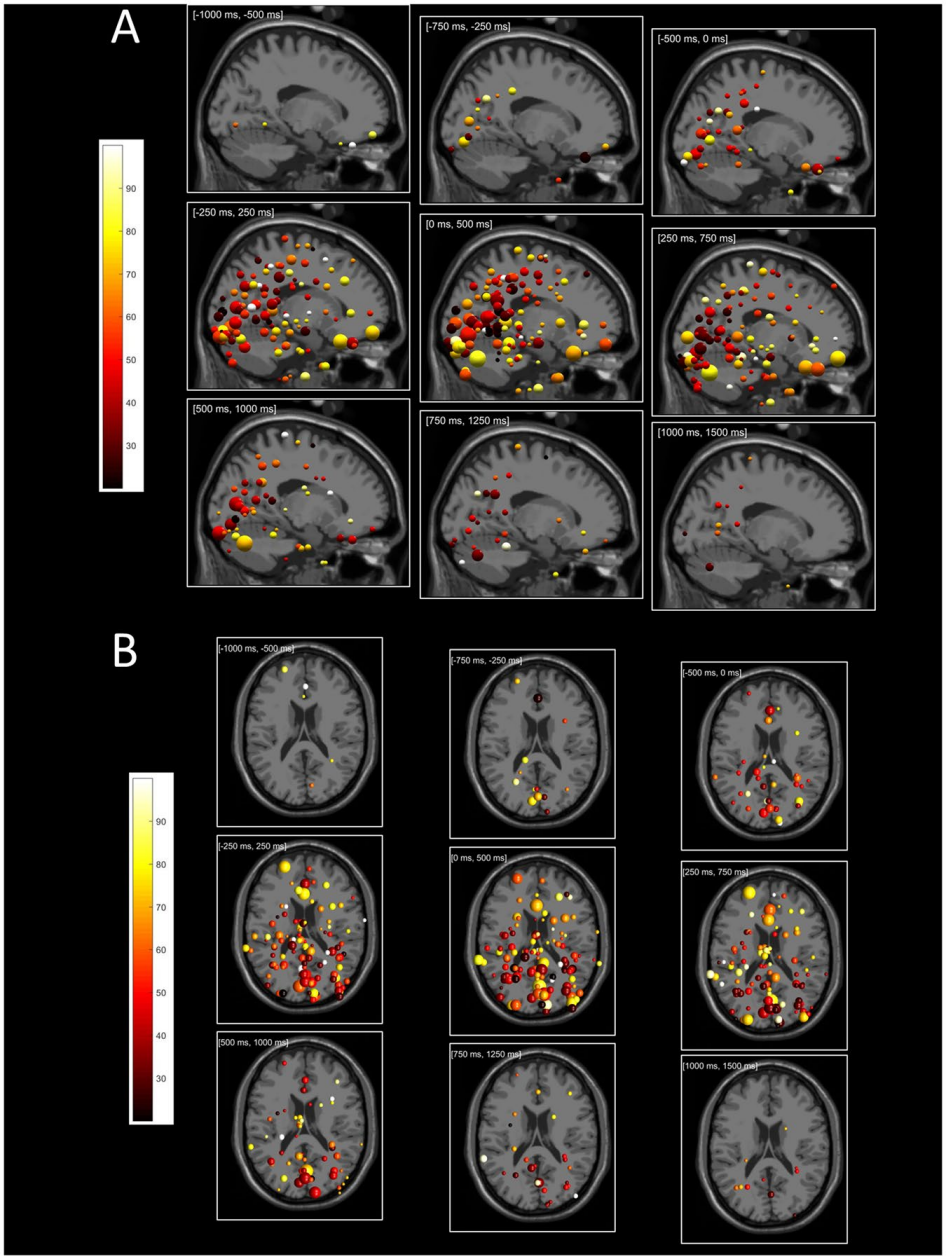
(**A**) Time-resolved directional tuning from [−1000 ms, −500 ms] to [+1000 ms, + 1500 ms] windows from (**A**) sagittal and (**B**) top perspectives. Tuning width for each component sphere is color coded as in the color bar. The logarithm of tuning height is represented by the component sphere radius. The range of significant (p < 0.05) tuning heights was from 4 × 10^−3^ (smallest) to 8 × 10^−2^ (largest).

Figure 6 illustrates direction tuning of ICs at their source locations from sagittal (Panel A) and axial (Panel B) perspectives (see also Supplementary Movie 1). The size and the color of the spheres indicate the log heights and tuning widths of the Gaussian-fit tuning curves. There is essentially no directional tuning in the −1000 ms to −500 ms (baseline) interval; directional tuning emerges in the −750 ms to −250 ms interval including both visual cue and movement onset. Directional tuning is strongest (in number of ICs and tuning magnitude) in the 0 ms to 500 ms interval, and disappears gradually as the movement finishes. We remark that directional tuning found in the perimovement periods (Fig. 4) were not by statistical chance as the same analysis found almost no directional tuning before movement onset and after movement end (Fig. 6).

### Shifts of directional tuning across workspaces

**Figure 7:**
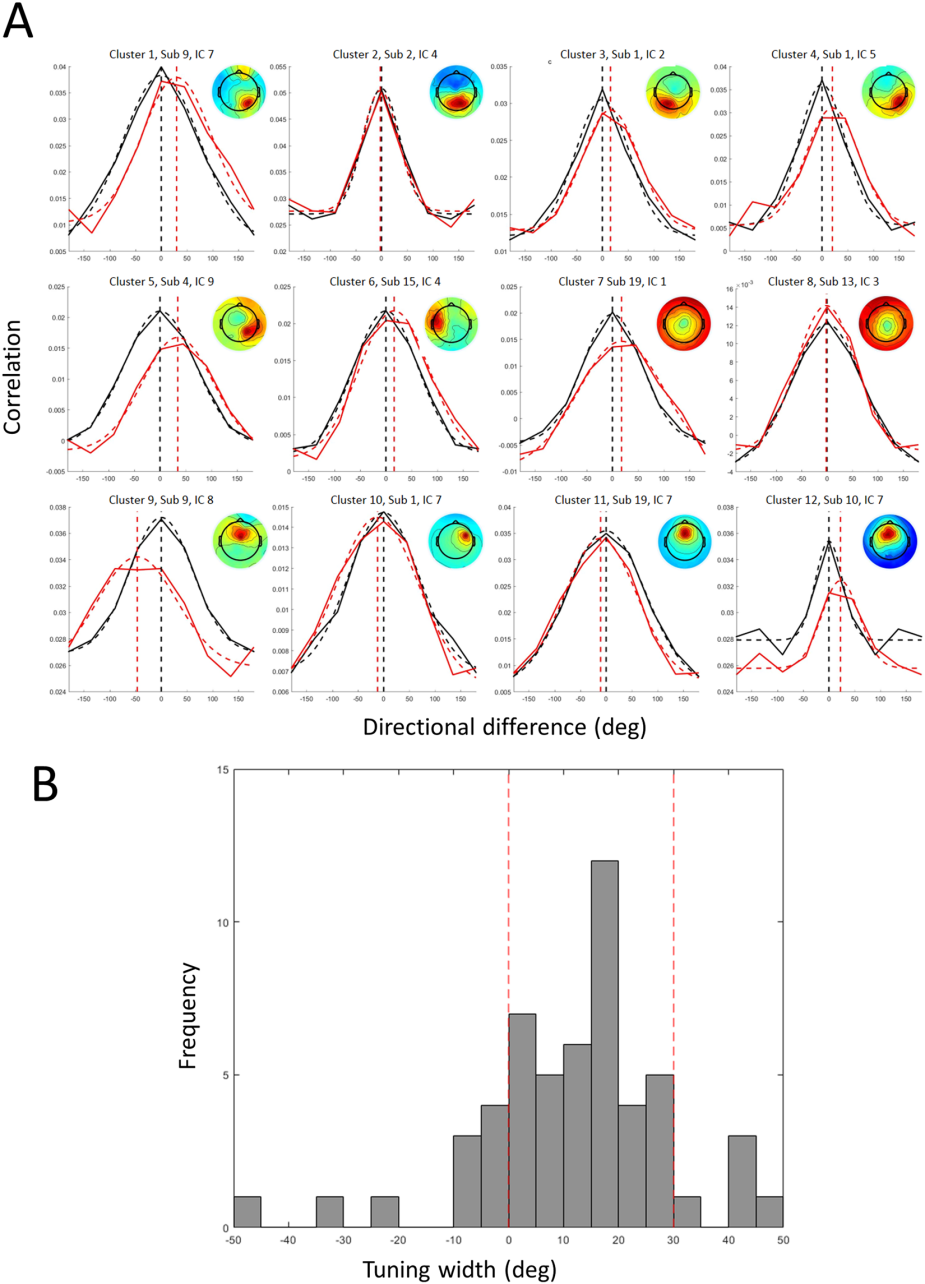
(**A**) Tuning shifts of representative components from twelve clusters. Tuning curves computed across the workspaces (red) are compared with those computed within one workspace (black), with a corresponding component map. (**B**) Histogram of directional tuning shifts from all components that exhibited statistically significant directional tuning.

**Figure 8:**
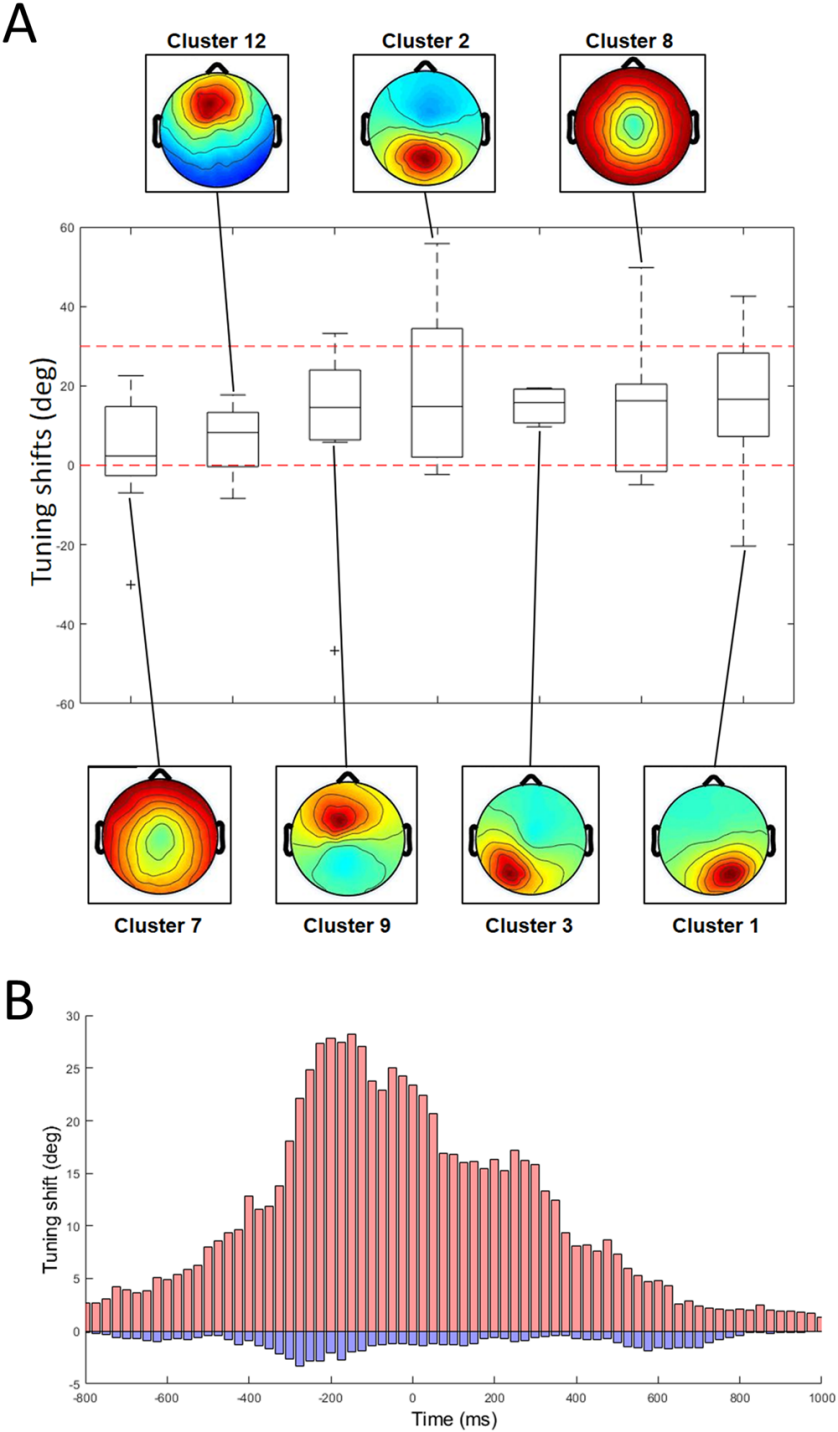
(**A**) Differences in direction tuning produced by moving from the right workspace to the left for twelve IC source clusters, sorted in an ascending order of the median value. Dashed red horizontal lines at 0° and 30° indicate differences consistent with external and shoulder-based reference frames, respectively. (**B**) Temporal changes of directional tuning shift from −1000 ms to 1000 ms. Each bin represents a weighted sum of tuning shifts multiplied with correlation amplitudes of dipoles computed in each time window. Positive (red bins) and negative (blue bins) shifts are illustrated separately.

We measured the shifts in direction tuning produced by moving from one to the other workspace using temporal correlations in the peri-movement latency window [−250 ms, 250 ms] for dipolar sources that exhibited statistically significant directional tuning within at least one workspace (*Computation of reference frames across two workspaces* in *Methods*). Of 108 dipolar sources with significant directional tuning within single workspace selected in the time window of [−250 ms, 250 ms] in the previous section, 69 exhibited direction tuning that was well fit to a Gaussian in both within-workspace and between-workspace measures. Direction tuning differences between workspaces peaked away from the (0°) origin as seen for exemplar direction-tuned ICs from each cluster (Fig. 7A). While direction tuning differences were widely distributed (ranging from −50° to + 50°), most shifts (70.9%) produced by moving from the right to the left workspace were positive, ranging from 0° to +30°, consistent with an extrinsic-like reference frame (no shift) or a shoulder-based reference frame (+30° shift), respectively (Fig. 7B). A cluster-by-cluster analysis of directional tuning shift is presented in Fig. 8A. In contrast to our finding that directional tuning widths tended to change along the anterior-posterior axis, we did not confirm any tendency of tuning shifts along the anterior-posterior axis.

**Figure 9:**
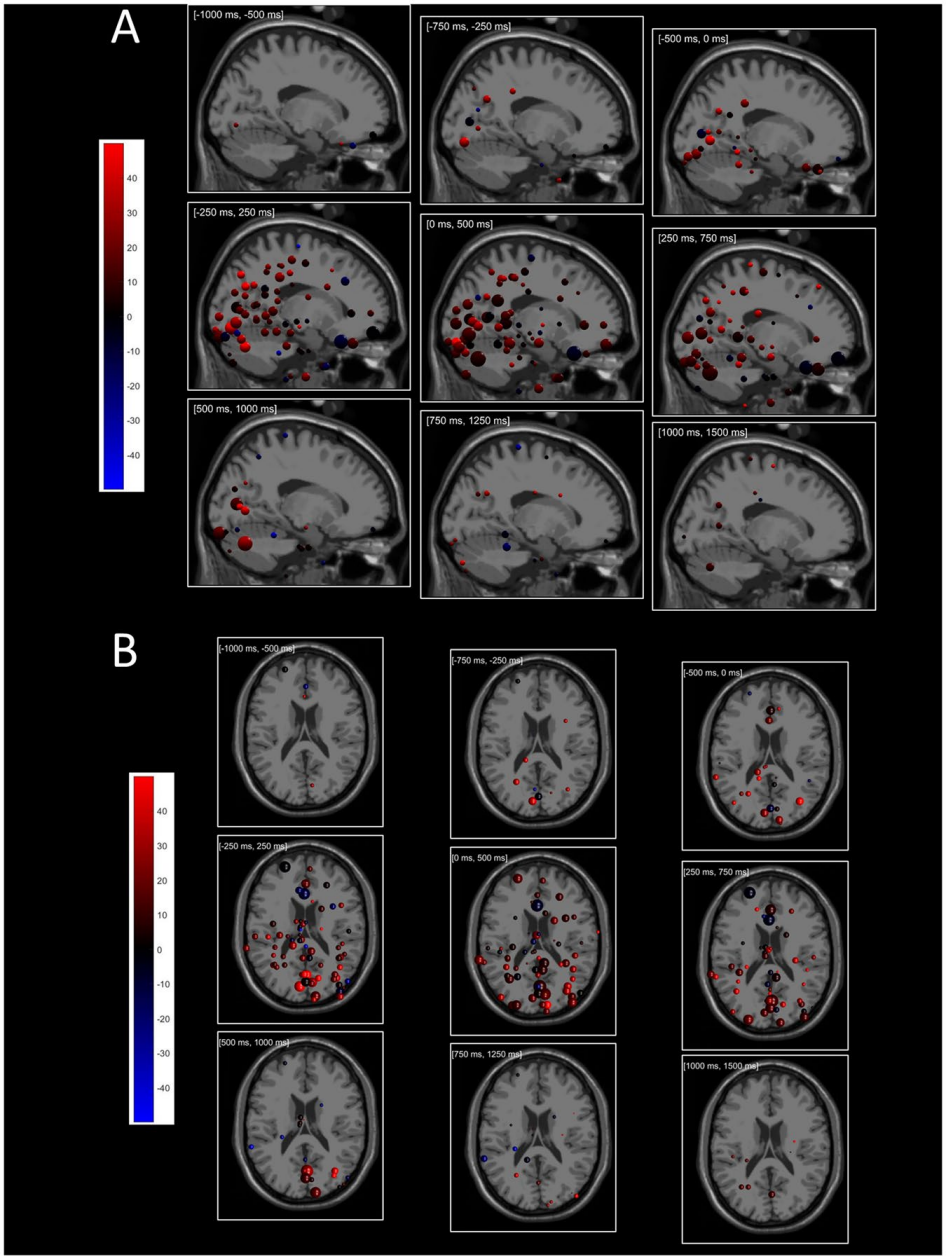
(**A**) Time-resolved tuning shifts in the [−1000 ms, −500 ms] to [+1000 ms, +1500 ms] windows from (**A**) sagittal and (**B**) axial perspectives. Tuning shift is color coded as in the color bar; log tuning height is represented by sphere radius. The range of tuning heights represented is from 0.004 (smallest) to 0.1 (largest).

Next, temporal dynamics of tuning shifts was computed in the same sliding windows. We characterized the degree of tuning shifts in each time window by computing a weighted sum of tuning shifts multiplied with tuning amplitudes (Fig. 8B). The weighted sum was computed separately for positive and negative shifts. Similarly as in the case of directional tuning, the degree of tuning shifts exhibited a peak around the perimovement period, and the positive shifts were dominant over all time windows. Figure 9 illustrates the temporal dynamics of tuning shifts from sagittal (Panel A) and axial (Panel B) perspectives (see also Supplementary Movie 2). In the peri-movement time window [−250 ms, +250 ms] most dipolar sources showed positive shifts (red) over the entire time course, and ICs with negative shifts (blue) had less Gaussian magnitude than those with positive shifts. As in the case of directional tuning, shifts in directional tuning across the workspaces dynamically appeared before movement onset and disappeared thereafter. This concludes that directional tuning of dipolar sources shifted mostly in a positive direction but not in the negative direction, consistent with the change in the shoulder angle and therefore consistent with representation of movement in a shoulder-based reference frame.

## Discussion

By exploiting recent advances in Mobile Brain/Body Imaging, this study employed high-density EEG and advanced signal processing methods to discover directional selectivity in the activities of EEG sources widely distributed through cortex. Further, their sensitivity to movement direction exhibited posture dependence, suggesting modulation of cortical potentials by variables representing movement planning and/or execution in either or both external (world) centered and body (shoulder) centered reference frames. The results of our exploratory approach implied that directional selectivity and associated representations of reference frame were not limited to classically-defined sensorimotor areas but could extend to the whole brain, to the contrary of our initial expectation.

A number of experimental paradigms have been used to investigate animal motor behavior, and neural mechanisms underlying motor control have been reported, as in the case of reaching movements reviewed in Introduction. However, there is still a gap between animal electrophysiology and human neuroimaging. Human fMRI experiments are incompatible with many motor tasks because of the susceptibility of the BOLD signal to movement artifact and its low temporal resolution. We thus hope our proposed method combining simultaneous recording of high-density EEG and body movements opens a new approach to understanding brain mechanisms of motor control and learning noninvasively, in natural settings with human subjects^23^.

### EEG studies

Several human EEG studies have examined features co-varying with the kinematics of upper limb^16–18,20,24,25^, finger^26–28^ and whole-body movements^29^. A recent study examining electrocortical dynamics of humans in a reaching task EEG experiment involving various reach magnitudes and speeds reported that theta band cortical field activity in the contralateral motor area covaried with arm movement kinematics^30^. However, their task involved arm/hand movements in only one direction; directional selectivity of EEG sources was not examined. Another study reconstructed hand movement velocity profiles in three-dimensional space by linear regression on scalp EEG data measures, identifying cortical source densities informative for kinematic reconstruction in precentral and postcentral gyri and inferior parietal lobule^31^, indicating that the information about movement velocity is encoded in scalp EEG time courses. Subsequent studies have reported decoding of movement kinematics from raw EEG time courses^16,18,26–29^ and from cortical source currents^20,25^ using mainly low-frequency features^24^. Therefore, we expected that EEG source activity time courses would contain information about movement direction. The previous studies were, however, not designed in comparison with monkey electrophysiological studies, and did not address the reference frames problem by employing movement tasks involving multiple workspaces or postures.

### fMRI studies

Previous human fMRI studies have reported directional tuning in primary motor cortex^10–13^, premotor cortex^10,12^, supplementary motor area^10^, and posterior parietal cortex^12^. In addition, in a visual rotation task, cursor movement directions in visual coordinates could be decoded from activities in visual areas, while actual hand movement directions could be decoded from activities in motor areas^32^. Compared with these fMRI studies, the strength of our study is our use of an task paradigm basically identical to earlier original monkey experiments, including in the magnitudes and kinematics of the reaching actions. We could also resolve, in the dynamics of widely distributed cortical EEG source areas, direct electrophysiological correlates of reaching actions not found in fMRI studies. Whereas spike-rate monkey studies show that the reference frames used for target location in parietal reach area and Brodmann Area 5 do not evolve over time^33^, directional tuning of single neurons exhibits dynamic changes, peaking for movement directions that vary before versus during the movement as reported for dorsal premotor cortex^34^ and for Purkinje cells in the cerebellum^35^; these dynamic features cannot be measured with BOLD signals having a time constant on the order of seconds.

### Electrophysiological studies

Previous electrophysiological studies of monkeys have shown that while a majority of neurons in motor-related areas are directionally tuned, tuning widths vary considerably, even within a single cortical area^36,37^. Furthermore, shifts in directional tuning in response to change in body posture or workspace also exhibit varying values, even in primary motor cortex^8,38^, indicating that dynamics consistent with use of multiple reference frames may coexist within one cortical area. However, directional tuning widths are reported to differ from area to area as neuronal groups. Neurons in the cerebellum, for example, tend to be more broadly tuned to movement direction that those in motor cortex^39^. Also, an area-by-area comparison about reference frames suggest that the premotor cortex likely represents movements in an (extrinsic) world-centered reference frame^9^, whereas in primary motor cortex movements are mainly represented in an intrinsic) body-centered reference frame^8^. Therefore, even given the large variance in tuning properties of individual neurons, it is possible to define statistical and area-specific characteristics that distinguished the activity of a neuronal population in one area from those in other areas. The current study demonstrated that dipolar source activities in different cortical areas show directional sensitivity and posture dependence.

A number of monkey experiment paradigms have been developed to better understand the neural dynamics supporting motor control and motor adaptation. One such approach has been to impose a load on a participant’s hand and to then dissociate its kinematics (direction of movement) from its dynamics (direction of force)^40–42^. This paradigm differentiated activity in primary motor cortex reflecting change in force direction^41^ from activity in parietal area BA5 regardless of load^42^. Another paradigm imposes a velocity-dependent force field on the hand during reaching and observes learning-related activity features in primary motor cortex^43^; this has been used to report on neurons whose spike rates maintain a memory of motor adaptation. These paradigms have not yet been employed in non-invasive human neuroimaging, mainly because of the physical constraints of fMRI experiments. Our approach is free from severe physical constraints; the results presented here demonstrate that high-density EEG signals, resolved into cortical sources by ICA, contain detailed information about body movements. We therefore expect non-invasive human experiments can be used to study implicit movement representations and mechanisms supporting motor control and adaptation that have been hitherto studied only in invasive animal experiments.

### Limitations

The current experimental design was not able to dissociate an important confounding factor; the observed movement directional tuning could result from movement preparation and execution, from visual processing of target directions, and/or from eye movement control. Spike rates of neurons in the frontal eye field and dorsal prefrontal cortex, for example, express eye-movement-related features that are modulated by movement execution^44^ and preparation^45^, respectively. Therefore, directional tuning of dipolar sources in those or nearby frontal areas might originate from eye movements. Based on our casual observations during the experiment, the subjects tended to look at the center location before movement and made a saccade toward a target when the target appeared on the screen. Indeed, the ICs of eye movements exhibited directional tuning (Supplementary Figure 6). In addition, spike rates of neurons in the somatosensory area, the posterior parietal area, and the cerebellum exhibited directional tuning with respect to a reaching target, as reviewed in Introduction. Directional tuning found in the different areas may have specific functional roles such as visual processing, visuomotor transformations or sensorimotor integration^46^. Our analysis of the time courses revealed that the four clusters exhibited peak values at different timings, thereby indicating their specific functional roles in pointing movements. But our current experimental design and analysis do not allow definitive interpretations of their functional roles. A possible way to dissociate these potential confounding factors is to design an experiment in which the timing of movement onset is strictly controlled. Inserting random-delay pauses in execution of intended movements, for example, could separate features relating to motor preparation from features relating to motor execution, often used in monkey electrophysiological studies. Systematic control of gaze direction during reaching could also separate eye- and head-centered representations^47,48^. These behavioral controls should be addressed in a future study.

In general, there is an issue of muscle artifact contamination in EEG studies of motor control. Previous studies reported that EMG signals contain noise components attributable to the electro-chemical noise at the skin-electrode interface^49^ and mechanical disturbance^50,51^ and are modulated by movement-related parameters such as speed of isometric force generation^52^ and velocity of the head and neck^53^. Other studies reported EMG signals whose power spectrum densities resembled those of scalp EEG channels^54,55^. Therefore, it is of importance that muscle artifacts were appropriately removed before analyzing the direction tuning in our study. We have used multiple criteria for the procedure of muscle artifact removal: power spectral densities, dipole positions, and scalp topography. Therefore, even if some of muscle artifact components had spectral characteristics similar to those of cortical sources, they should have been removed by the location and scalp-topography criteria. It is still conceivable that ICA separation was not perfect and that some independent components contained both cortical and muscle components. This possibility is biologically inevitable because cortical sources and muscle component are coherent in the frequency space^54^.

## Methods

Our analysis procedure involves motion data analysis, EEG data analysis of individual subjects, EEG data analysis of the subject group, and directional tuning analysis of all independent components, which is summarized in Supplementary Figure 1.

### Subjects

Nineteen subjects with normal or corrected vision participated in a pointing experiment (12 males and 7 females, ages: 19–44 (mean 24.4 ± 6.8 SD)). None of them reported any history of neurological conditions. One subject was left handed; the others were right handed. Each subject signed a written informed consent form approved by an Institutional Review Board (IRB) of the University of California San Diego. Separately, informed consent to publish videos and photos taken during the experiment was obtained from each subject. The experiment was designed and performed under the IRB board’s appropriate guidance and based on Declaration of Helsinki.

### Pointing task

Subjects performed a center-out pointing task toward one of eight targets arrayed in one of two circular workspaces, near-identical to those used in previous studies of monkey electrophysiology^1,6,7,38,56^. Subjects were seated in front of a 17-inch LCD display laid horizontally at about the height of their navel, their body midline facing the center of the display screen. Two circular workspaces were shown in the left and right sides of the screen, their centers separated by 20 cm. To address the left and right workspaces, the subject’s shoulder needed to rotate approximately 30°. In the active workspace, a central cross indicted the movement starting position. Eight small gray disks (possible movement targets) were arrayed 45° apart in a 10-cm radius circle centered on the starting cross (Fig. 1A).

At the beginning of each pointing trial, the subject was asked to place their right index finger on the cross shown on the display. A red arrow indicating one of the eight target directions then appeared at the center of workspace (‘Cue event’), and after 500 ms the corresponding target disk turned red (‘Go event’), prompting the subject to move their arm and hand so as to point at it with their right index finger. After 500 ms, the target returned to gray (‘Back event’) prompting an inbound movement back to the starting position. The Cue, Go, and Back events were accompanied by (beep) sounds, and the subjects were asked to hold their movements until they heard the expected Go or Back beep.

Thus in each trial subjects were instructed to make outbound pointing movements from a starting position to one of eight targets and then an incoming return pointing movement from the target to the starting position, and to make sure to stop moving during target pointing so that their movements were not continuous but discrete. Each block consisted of 240 trials (30 pointing movements per target) lasting about eleven minutes. The order of the presented targets was pseudo-randomized. The workspace used was switched after each block, the workspace order counterbalanced across subjects. Two subjects performed eight blocks of trials (four blocks in the right and left workspaces, respectively); the other ten subjects performed six blocks (three blocks in the right and left workspaces, respectively). No specific instructions about eye movements were provided, and subjects’ eye movements were not monitored.

### Simultaneous recording of high-density EEG and body motion

During the pointing task, EEG signals were recorded using 205 electrode channels distributed across the scalp using a BioSemi Active II amplifer (Amsterdam, Netherlands) (Fig. 1A). Before the experiment, the three-dimensional positions of the electrodes were measured using a digitizer (ELPOS, zebris Medical GmbH, Isny im Allgau, Germany) to allow subsequent equivalent current dipole source localization. In addition, body movements were measured using nine active LED emitters in a room motion capture system (PhaseSpace, San Leandro, CA), placed on the subject’s head, shoulder, upper arm, forearm, wrist and index finger. The EEG and motion capture sampling rates were 512 and 480 Hz, respectively. The recordings of EEG and body movements were synchronized with sub-millisecond precision using the open source Lab Streaming Layer (LSL) software framework (*github*.*com/sccn/labstreaminglayer/)*. The combined EEG and motion tracking data were stored in the Extensible Data Format (XDF) (*github*.*com/sccn/xdf/*) and were imported into Matlab for processing using the MoBILAB toolbox^57^ along with the latencies of Cue, Go, and Back events sent to the LSL data stream by the custom experiment control script written in Python.

### Motion data analysis

The motion data of the right index finger was used to detect pointing movement onset; other motion capture data were not used for this report. An outbound pointing movement of the index finger from the central cross to a target disk, recorded in every trial, was smoothed using a 10-sample (20.8-msec) moving average. The smoothed trajectory was then numerically differentiated to compute velocity tangential to the display. A speed profile (velocity norm) was then computed and maximum speed for each trial was recorded. Movement onset and offset were defined as the latencies at which movement speed respectively first exceeded or fell below 5% of the maximum speed in that trial. Trial movement duration was defined as the time interval between movement onset and offset. Movement onsets and offsets were used to extract movement-aligned EEG signals in further processing. We excluded from further analysis trials in which movement onset was more than 300 ms before or after the Go event latency.

### EEG preprocessing

EEG signals were preprocessed and analyzed using the open-source EEGLAB signal processing environment^58^. Preprocessing consisted of down sampling, high-pass filtering, line-noise removal, and artifact subspace reconstruction (ASR). EEG signals were first down sampled to 256 Hz to reduce disk storage requirements and processing time and then high-pass filtered using a basic FIR filter (cutoff frequency 0.5 Hz, transition bandwidth 1 Hz, Hamming windowed). A 1-Hz high-pass filter suppresses low-frequency drifts that are spatiotemporally nonstationary and helps ICA produce a good decomposition^59^. Line noise at 60 Hz and its second harmonics at 120 Hz were removed by the CleanLine EEGLAB plug-in which adaptively estimates and removes sinusoidal artifacts using a frequency-domain regression technique^60^. Another plug-in, *clean_rawdata*, removed outlier channels. ASR was then used to detect and subtract non-stationary high-amplitude artifacts that most probably originated from eye blinks, muscle, and electrode motion and to interpolate or reject periods in the EEG data that exceeded by 4 standard deviations of the mean amplitude of a clean portion of the same data^61^. Supplementary Figure 2 illustrates how ASR removed non-stationary and high-amplitude artifacts for a representative subject.

### Independent component analysis

Our goal was to explore directional selectivity and reference frames over the brain without restricting to sensorimotor areas, so EEG sources were decomposed by independent component analysis. After preprocessing, EEG signals were decomposed into temporally maximally independent component (IC) processes using Adaptive Mixture Independent Component Analysis (AMICA)^62^, provided as an EEGLAB plug-in at *sccn*.*ucsd*.*edu/~jason/amica_web*.*html*. Unlike other ICA algorithms such as extended infomax that assume fixed super- or sub-Gaussian distributions for signal sources, AMICA models IC source distributions as a mixture of generalized Gaussian functions. In a recent assay, AMICA outperformed 21 other algorithms in reducing the mutual information among the returned ICs^63^. In the present study each source distribution was modeled as a mixture of three generalized Gaussians. Using the three-dimensional locations of the EEG electrodes measured for individual subjects, corresponding scalp topographies (columns of inverse of the unmixing matrix) were plotted^64^. From the continuous time courses of the IC processes, 3-sec trial epochs were extracted from 1 sec before to 2 sec after pointing movement onsets. For each epoch, a baseline value, the mean of the pre-movement period, was then subtracted from the epoch.

### Estimating equivalent current dipoles for EEG sources

Once IC activities and scalp projections were obtained, the location and orientation of a best-fitting equivalent current dipole was fit for each component using the DIPFIT2 plug-in for EEGLAB, derived from the open source software fieldtrip^65^. As MRI structural head images of the subjects were not available, a boundary element model based on the MNI standard brain template was used for estimation of dipole locations and orientations. Many of the IC scalp projections were “dipolar” (dipole-like) meaning that the component map is well modeled as the projection of a single equivalent current dipole^66^. ICs whose scalp projection (scalp map) well fit the projection of a single equivalent dipole are hereafter referred to as ‘dipolar’ sources. To simplify the data processing, a single equivalent dipole model was estimated for each IC, although a small number of ICs exhibited bimodal scalp maps.

### Independent component selection

ICs obtained by ICA decomposition (usually the same number as EEG channels), represent information sources of the data, not only components of brain origin but also artifacts of non-brain physiological origin (eye movements, eye blinks, muscle activities, or cardiac pulses) or mechanical origin (loose electrode noise, electric line noise). Fortunately, these non-brain ‘artifact’ components have characteristic time course, power spectral, component map and dipole location features distinct from those originating in the brain. There are several published criteria for the selection of artifact components^67,68^. First, artifacts originating muscle activities were identified on the basis of spectral features (resembling electromyographic activity). Templates of power spectral densities of muscle activities used for this identification are summarized in Supplementary Figure 3. Second, saccade and eye blink artifacts were manually identified by their characteristic component scalp topography features. Third, artifact components accounting for unusual channel noise were identified by their equivalent current dipole locations being located out of the brain volume. Finally, artifact components whose residual variance in the scalp topography, compared with noiseless projection of the estimated equivalent current dipole, showed >15% were identified^69^. Components that survived identification by the above criteria were submitted to the final analysis.

### Group analysis

All dipolar sources from individual subjects were assembled for a group analysis using the STUDY framework in EEGLAB^70^. The group analysis aimed to statistically aggregate similar ICs from all subjects according their characteristics. Dipolar sources from all subjects were clustered according to their three-dimensional locations and spectral features using the standard *k*-means algorithm^69^. Feature vectors consisted of positions of dipoles (three dimensions) and spectral features ranging from 3 Hz to 50 Hz (ten dimensions); relative weights for dipole position and spectra were set to 10 and 1, respectively. The feature vectors were then reduced to ten dimensions using principal component analysis. The procedure of dimensional reduction guarantees a better performance of *k*-means clustering algorithm. The number of the IC clusters is an open parameter and needs to be determined by empirical tests. First, we set it a rule that minimum 50% of unique subjects should be included in each cluster. This limited the maximum number of clusters to be fifteen. Second, we varied the number of the clusters to evaluate stability of the results, and confirmed that the results were stable from ten to fifteen. To balance the spatial resolution and the number of minimum unique subjects, we determined that the number of IC clusters was twelve. Each cluster contained 71.9% (SD 9.6%) of subjects, and the minimum ratio of unique subjects per cluster was 52.6% (see Table 1). Average power spectral densities of the twelve clusters showed 1/f type curves (Supplementary Figure 4), which clearly differed from those of muscle activities (Supplementary Figure 3), indicating that our procedure of IC selection functioned properly. For all the ICs in the twelve clusters, the computation of directional tuning was then performed as described below.

### Computation of directional tuning within a single workspace

Each IC of scalp EEG represents synchronized field activity in an area of neuropile containing at least hundreds of thousands neurons. In contrast, directional tuning and reference frames have been measured as properties of single neuron firing rates in monkey electrophysiology experiments. We expected it to be difficult, if not impossible, to make a direct comparison between EEG and single-neuron spike rate data. Thus we proposed a workaround – to compare the EEG time courses during pointing movements in one direction with those for movements in other directions. We hypothesized that the EEG activity associated with movements in one direction should more resemble that for adjacent directions given the broad directional tuning expected of a large neural population. As the pointing direction becomes more distant, the resemblance of the EEG epochs should decrease. Based on this intuition, the degrees of similarity in component activities for pairs of movement directions was evaluated by computing temporal correlation coefficients between EEG time series for the two movement directions. As the movements in different directions were not concurrent, we averaged correlations between all possible pairs of epochs captured during movements in the two directions.

Similar analyses have been performed in fMRI studies in which similarities of responses to visual stimuli were evaluated according to the similarity of BOLD signals following presentations of those stimuli; this has been termed representational similarity analysis (RSA)^71–74^. All the ICs in the twelve clusters after group analysis were submitted to RSA. Our analysis employed RSA to evaluate the direction sensitivity of each IC. As a measure of similarity, Pearson correlation coefficients between pairs of EEG time courses accompanying pointing movements in two directions were computed using a moving time window. The analysis window was from −1000 ms to 1000 ms surrounding movement onsets, on which 500-ms square-window smoothing was applied with a 25-ms step size. A correlation coefficient *r*_*i*j_ between all pairs of EEG time courses time locked to movements in direction *i* and direction *j*, respectively, was computed. Thereby an 8 × 8 matrix was constructed whose (*i*, *j*) entry represented the correlation coefficient between EEG during movements in directions *i* and *j* (Fig. 3A). This matrix was computed first separately for movements in the left and right workspaces, and then averaged across workspaces. To obtain a directional tuning curve, eight rows of the matrix were aligned according to movement direction difference (from −180° to +180°) and then averaged (Fig. 3B). Note that the tuning curves are symmetric because correlation coefficients were symmetric about indices *i* and *j*. These directional tuning curves were fit by a Gaussian function parametrized by three parameters (standard deviation *σ*, height *a* and baseline *b*).

$aexp(-\frac{{x}^{2}}{2{\sigma }^{2}})+b,$ where *x* denotes the movement direction difference in degrees and *σ* quantifies the width of directional tuning. We selected components according to two independent criteria: fit to the Gaussian function and the height of directional tuning (see Statistical Testing).

As a validation of our method of computing directional tuning, we have applied RSA to the muscle ICs which were rejected by a criterion of power spectral densities (see “Independent component selection”). It is known that EMGs have cosine-like broad directional tuning in wrist movements^75^, so we expected that RSA should recover directional tuning also in our data. We found that many of muscle ICs exhibited broad directional tuning (see several examples in Supplementary Figure 4), thereby validating our application of RSA to the computation of directional tuning.

### Computation of reference frames across two workspaces

In monkey electrophysiological studies, the reference frame in which a neuron represents body movements has been studied by evaluating a shift in directional tuning curves across different workspaces or body postures^6–9,38,56^. Here, we computed correlations between EEG time courses of movement direction *i* in the left workspace and those of movement direction *j* in the right workspace. If such a correlation curve has a peak at the origin (0°), EEG time courses in corresponding directions in the two workspaces are most correlated, indicating alignment to an extrinsic reference frame. In contrast, if the peak of the correlation curve is shifted from the origin, this should indicate alignment to a non-extrinsic reference frame. Specifically, if the peak of the correlation curve is shifted by 30° (the shoulder rotation corresponding to the posture change across the workspace), this should indicate a shoulder-based reference frame. Similar to the case of single workspaces, an 8 × 8 matrix was computed whose (*i*, *j*) entries represented correlation coefficients between direction *i* in the right workspace and direction *j* in the left workspace. Eight rows of the matrix were sorted according to movement direction differences, averaged and then fitted with a Gaussian function, ${aexp}(-\frac{{(x-{x}_{0})}^{2}}{2{\sigma }^{2}})+b$ (Fig. 3C). Note that, in contrast to computation of tuning curves for one workspace, the tuning curves between the two workspaces were not necessarily symmetric and could have a peak away from the origin. The mean of the Gaussian, *x*_0_, quantified the shift in direction tuning between the workspaces. Shifts of tuning were computed only for components which were selected by statistical testing.

### Statistical testing

To assess their directional selectivity in the peri-movement time window [−250 ms, 250 ms], ICs were first selected on the basis of goodness of fit to a Gaussian and on its height *a*. First, we identified ICs whose directional tuning curves were modulated smoothly over movement directions. We selected ICs whose goodness of fit *R*^2^ after fitting to a Gaussian was above 0.90. Next, statistical significance of the maximum height of the fit direction tuning curves was determined by applying a nonparametric permutation test with maximum statistics method as a generalized family-wise error rate (FWER) correction for multiple comparison correction^76,77^. Our null hypothesis was that time course of each IC does not have directional selectivity and hence the directional tuning curve should be flat. The null hypothesis was represented by the surrogate data which was generated by randomly permuting eight labels of movement directions in computing the directional tunings, and measuring the heights of the fitted Gaussians. The iteration was repeated 5,000 times for all ICs, and the second maximum values across all the ICs for each iteration was stored to build distribution of the generalized maximum statistics. For the multiple comparison correction, 95-percentile value of the generalized maximum statistics was adopted as a critical value that can correct all IC’s uncorrected data for both workspaces. That is, if an IC had a fitted Gaussian that was higher than this omnibus correction criterion, the IC was regarded as showing statistically significant directional tuning. There is a recent issue of circular analysis in neuroimaging data analysis^78^. We here remark that the goodness of fit to a Gaussian function and the height of directional tuning are independent quantities. Hence the initial selection based on the goodness of fit and the subsequent statistical test are independent to each other.

The statistical test described above was computationally expensive, taking about 20 hours on a desktop computer for one time window, so it was not practical to iterate the same test for all time windows. We applied the same threshold computed in the time window of [−250 ms, 250 ms] to data of other time windows, just for the purpose of selecting ICs for visualization.

### Data availability

The combined EEG and motion tracking data were stored in the Extensible Data Format (XDF) (*github*.*com/sccn/xdf/*) and will be uploaded to our ftp server (ftp://sccn.ucsd.edu/pub/).

## Electronic supplementary material

Supplementary Video 1

Supplementary Video 2

Supplementary Materials
