# Supplementary material for: Dynamics of directional tuning and reference frames in humans: A high-density EEG study
Source: Sci Rep. 2018 May 29;8:8205. doi: 10.1038/s41598-018-26609-9 (PMC5974292; doi:10.1038/s41598-018-26609-9)
Supplement: Supplementary file 3 — Supplementary Materials [file 41598_2018_26609_MOESM3_ESM.docx]

**Dynamics of directional tuning and reference frames in humans: A high-density EEG study**

**Abbreviated Title**: Dynamics of directional tuning and reference frames in human EEG

*Hirokazu Tanaka* ^a*^, *Makoto Miyakoshi* ^b^, and *Scott Makeig* ^b^

a. School of Information Science

Japan Advanced Institute of Science and Technology

1-1 Asahidai, Nomi, Ishikawa 923-1292, Japan

b. Swartz Center for Computational Neuroscience

Institute of Neural Computation

University of California San Diego

9500 Gilman Drive # 0559

La Jolla CA 92093-0559, U.S.A.

**Corresponding author**: Hirokazu Tanaka

School of Information Science

Japan Advanced Institute of Science and Technology

1-1 Asahidai, Nomi, Ishikawa 923-1211, Japan

Email: hirokazu@jaist.ac.jp

Tel: +81-761-51-1226 Fax: +81-761-51-1149

**Supplementary Materials**

The supplementary materials contain additional seven figures: : a flowchart of our analysis procedure (Suppl. Fig. 1), an example of removal of non-stationary artifacts using the artifact subspace reconstruction (ASR) method (Suppl. Fig. 2), representative templates of power spectral densities (PSDs) of muscle artifacts (Suppl. Fig.3), PSDs of cortical components from the twelve clusters (Suppl. Fig. 4), directional tuning of representative muscle and eye artifacts (Suppl. Figs. 5 and 6), and centroid locations of the twelve clusters (Suppl. Fig. 7).


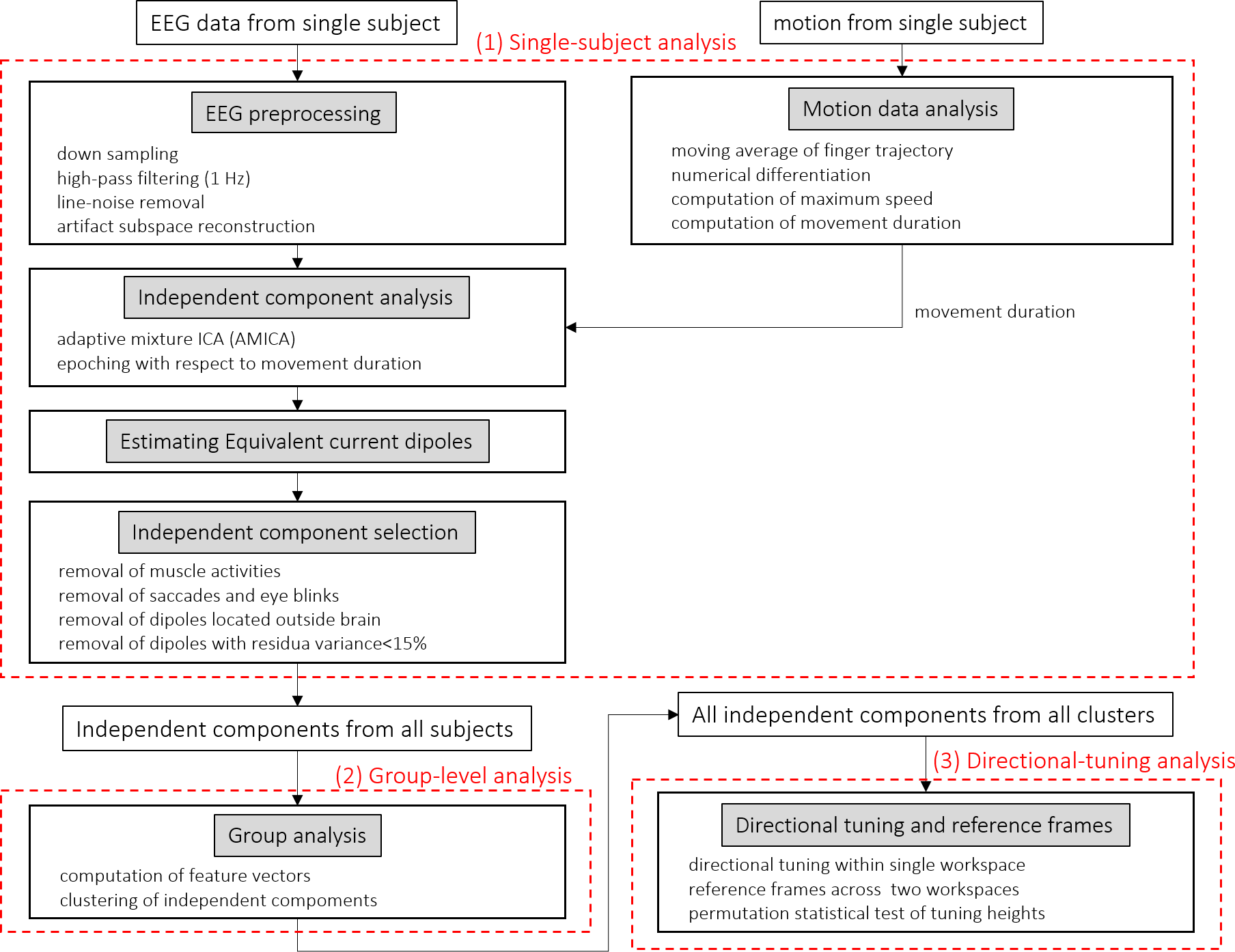


Supplementary Figure 1. Flowchart of our analysis procedure. Our procedure consists of (1) single-subject analysis step, (2) group-level analysis step, and (3) directional-tuning analysis step. Gray shades boxes correspond to the subsections in Methods.


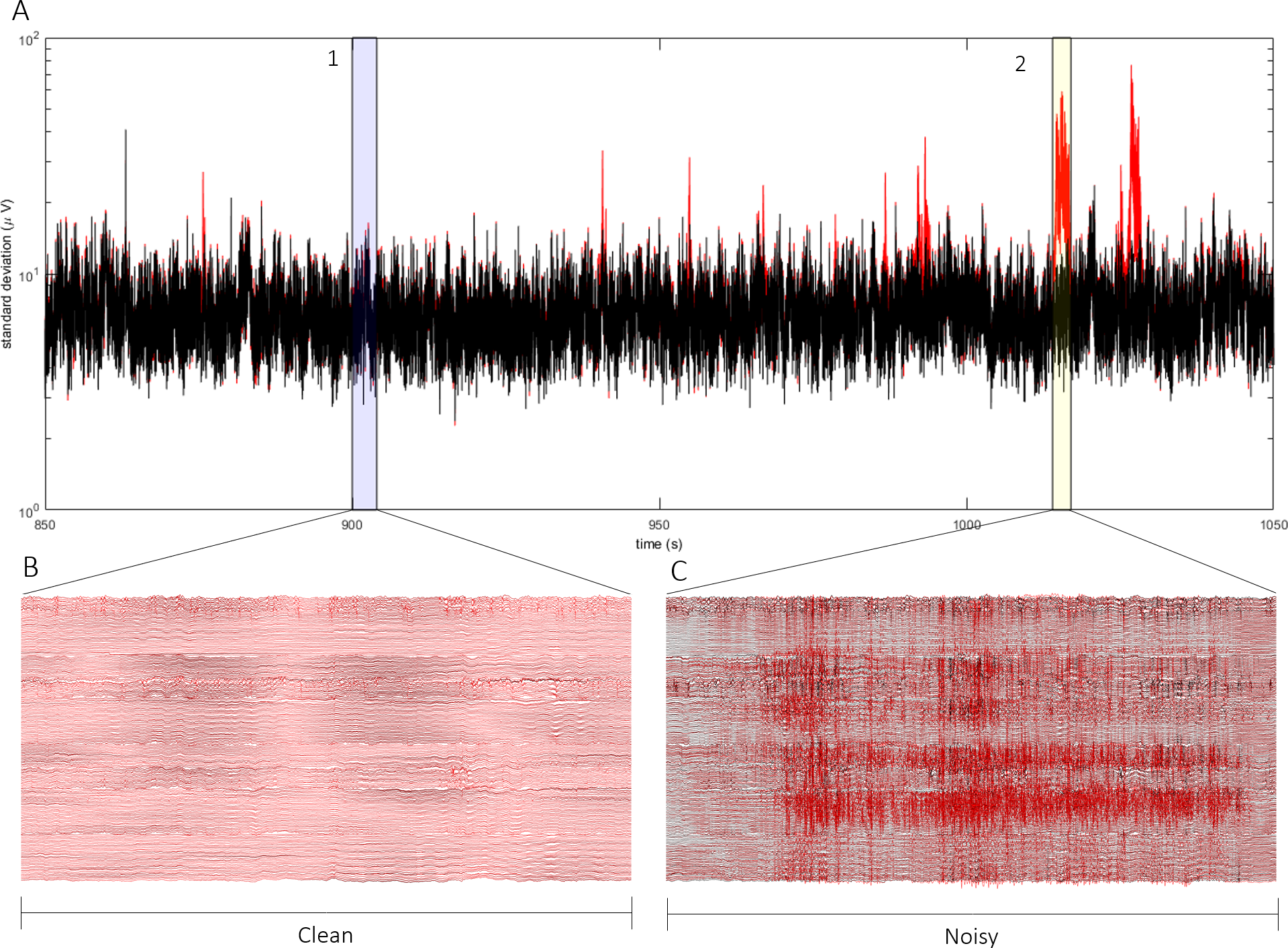


Supplementary Figure 2. Illustration of how ASR removes non-stationary, large-amplitude noises. For this subject, ASR rejected 0.02 % of the whole data, indicating that this data was relatively clean. ASR interpolated almost 100 % of the whole data and the variance was reduced by about 70% for the interpolated periods. (A) Time-by-time standard deviations of pre-ASR (red) and post-ASR (black) EEG data. In most of time, the two standard deviations overlap, while there are bursts in standard deviations of pre-ASR data. Note the ordinate in a logarithmic scale. (B), (C) Comparison between pre-ASR (red) and post-ASR (black) EEG data. In panel (B), a clean portion of 3 seconds (900-903s) was taken, and pre- and post-ASR EEG mostly overlap. In contrast, in panel (C), a noisy portion of 3 seconds (1014-1017s) was taken, and the large bursts of artifacts are corrected by ASR.


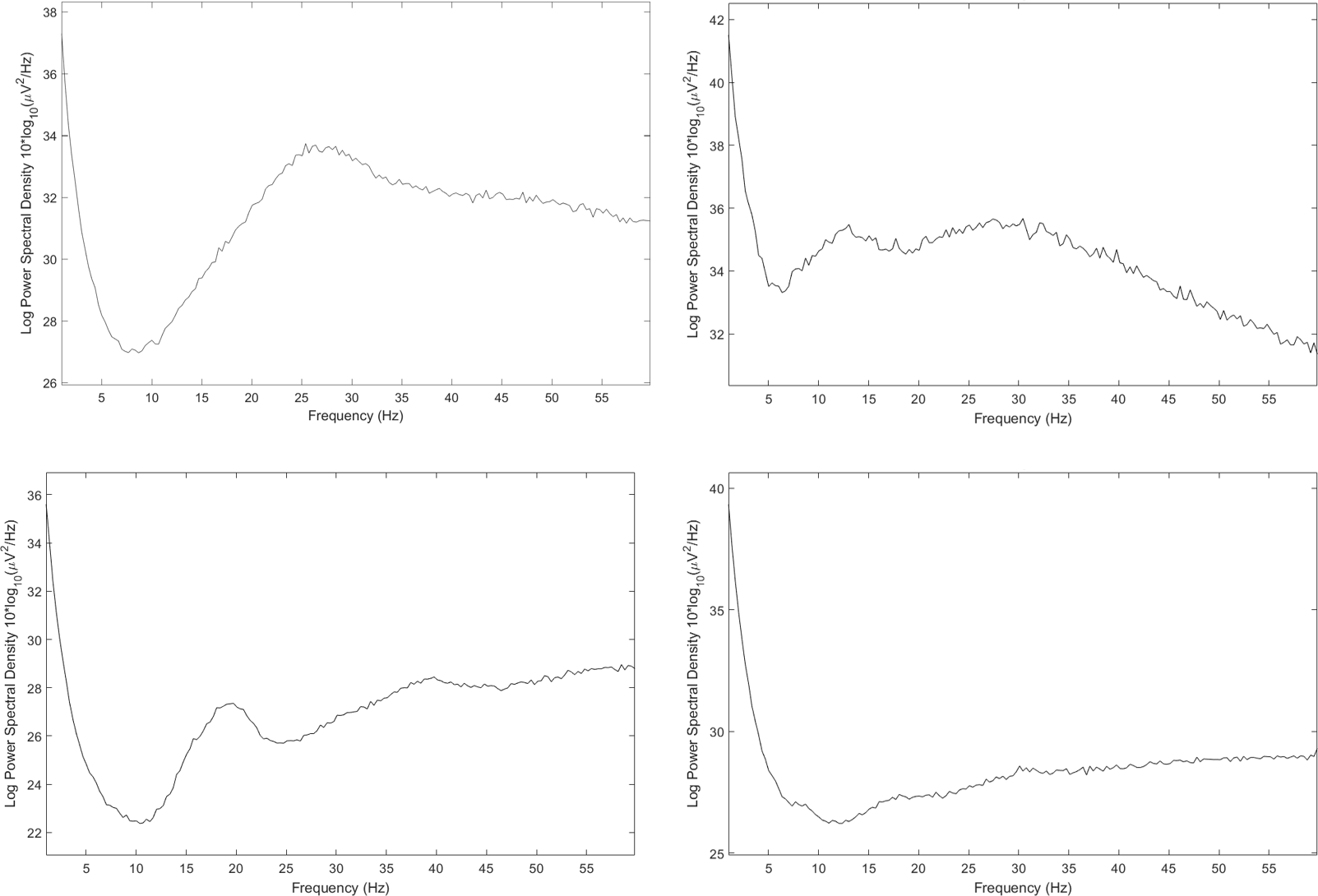


Supplementary Figure 3. Representative templates of power-spectral densities of muscle artifacts. Note that muscle artifacts were identified by using the power-spectral features of all frequency ranges. Muscle artifact components identified by the spectral features were removed and not analyzed for directional tuning.


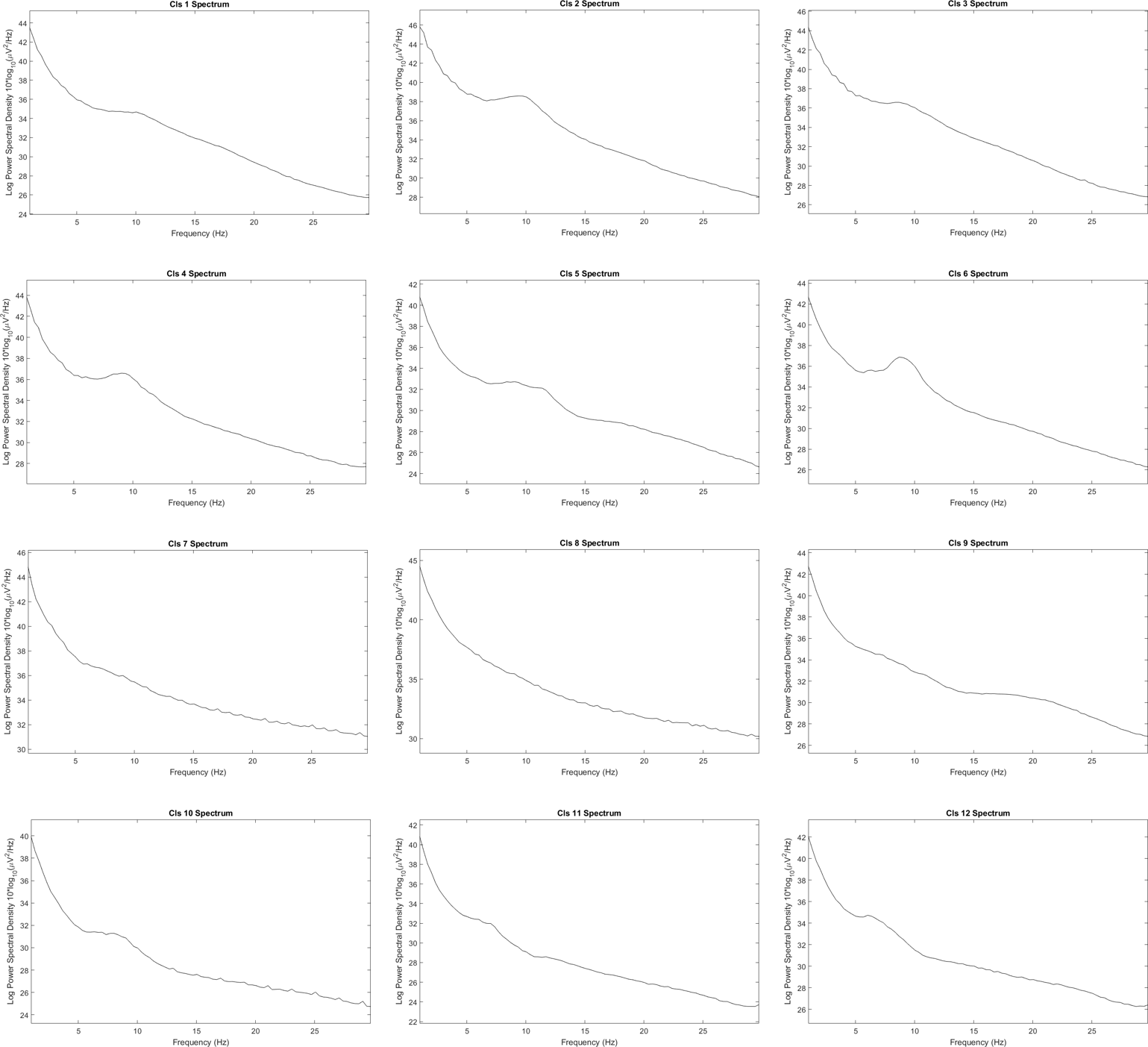


Supplementary Figure 4. Average power-spectral densities computed from the twelve clusters. All the densities show 1/f shapes characteristic of cortical sources.


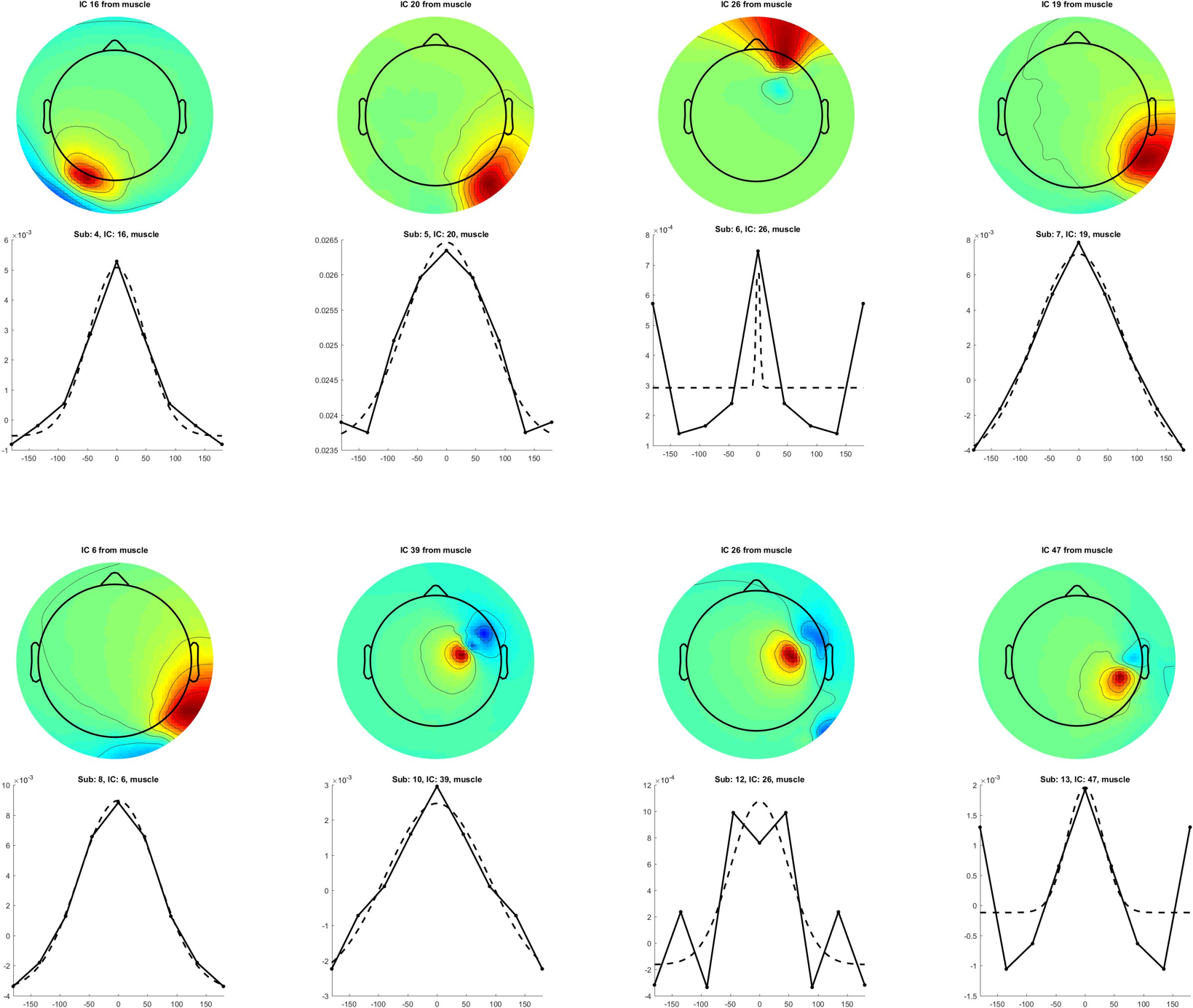


Supplementary Figure 5. Typical directional tuning of muscle ICs. These ICs were selected by a criterion of power-spectral densities illustrated in Suppl. Fig. 3. These components were not analyzed and shown in the main text.


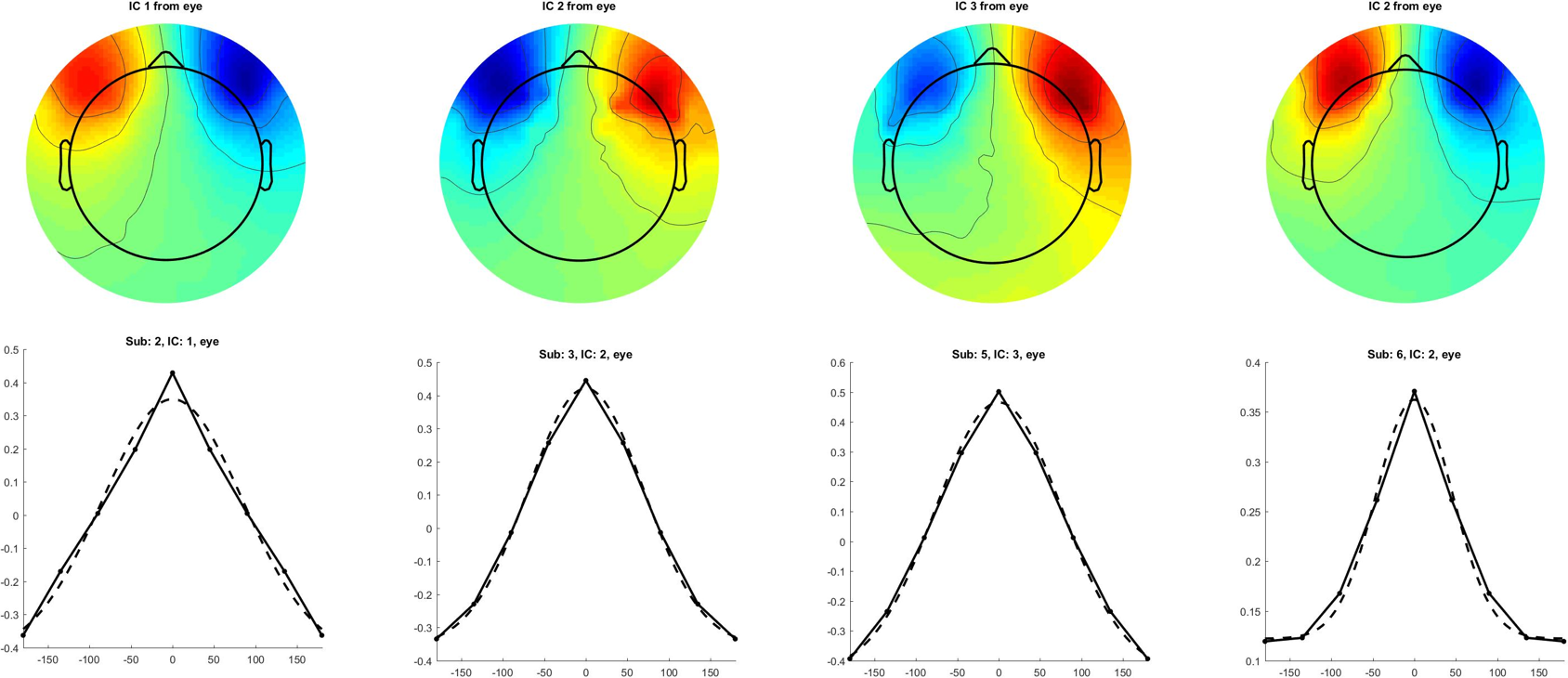


Supplementary Figure 6. Typical directional tuning of eye ICs. Note that the magnitudes of directional tuning are two magnitudes larger than those of muscle ICs.


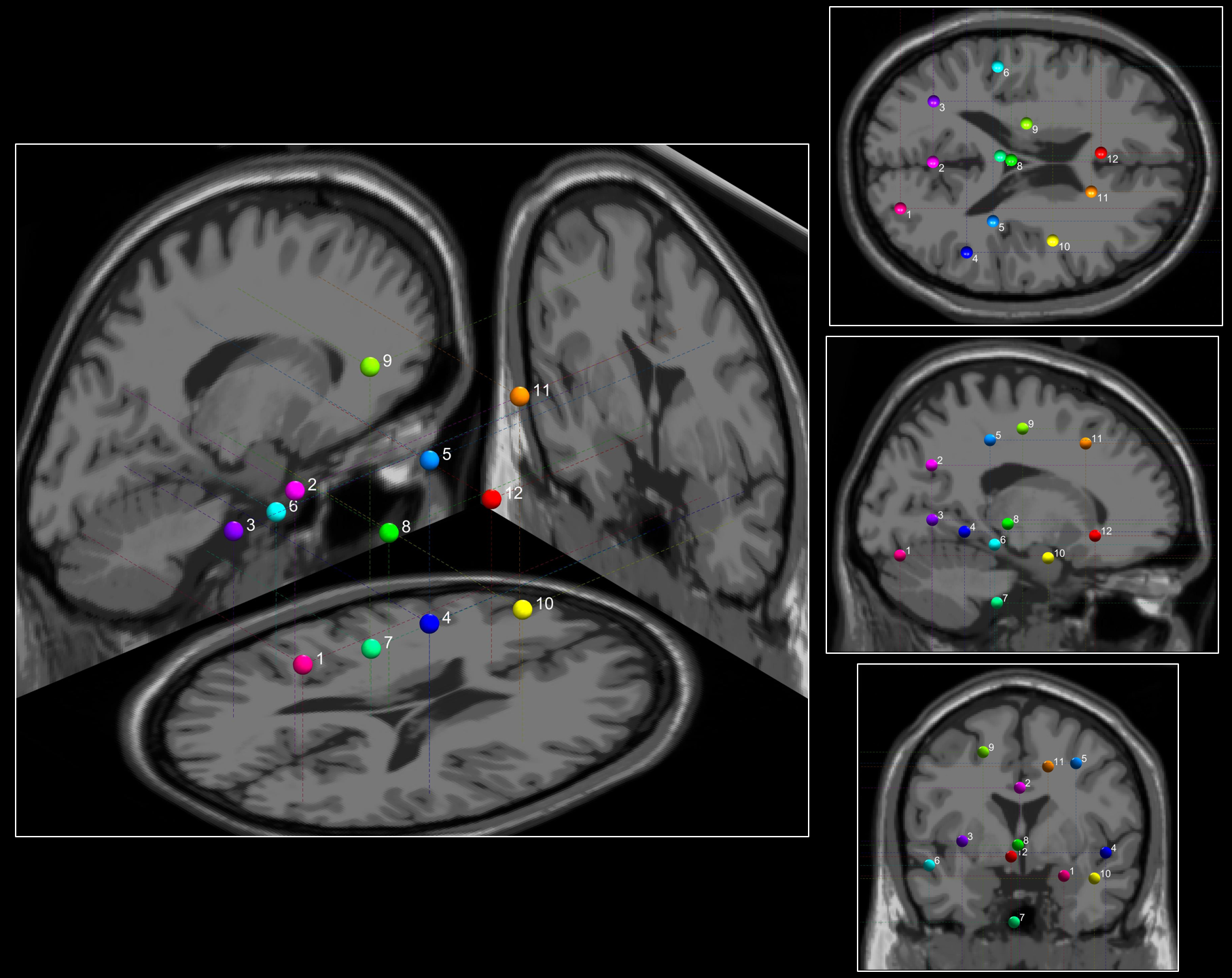


Supplementary Figure 7. Centroid positions of the twelve clusters (see the exact locations in Table 1 in the main text). The colors of the clusters correspond to those used in Fig. 2 in the main text.
